# Screening for frailty in primary care: a systematic review of the psychometric properties of the frailty index in community-dwelling older people

**DOI:** 10.1186/1471-2318-14-27

**Published:** 2014-03-06

**Authors:** Irene Drubbel, Mattijs E Numans, Guido Kranenburg, Nienke Bleijenberg, Niek J de Wit, Marieke J Schuurmans

**Affiliations:** 1 Department of General Practice, Julius Center for Health Sciences and Primary Care, University Medical Center Utrecht, Str. 6.131, Universiteitsweg 100, 3584 CG, Utrecht, the Netherlands; 2 Department of Public Health and Primary Care, Leiden University Medical Center, Hippocratespad 21, 2333 RC, Leiden, the Netherlands; 3 University Medical Center Utrecht, Department of Rehabilitation, Nursing Science and Sports Medicine, Heidelberglaan 100, 3584 CX, Utrecht, the Netherlands

**Keywords:** Frailty, Systematic review, Psychometric properties, Primary care, Screening, Older patients

## Abstract

**Background:**

To better accommodate for the complex care needs of frail, older people, general practitioners must be capable of easily identifying frailty in daily clinical practice, for example, by using the frailty index (FI). To explore whether the FI is a valid and adequate screening instrument for primary care, we conducted a systematic review of its psychometric properties.

**Methods:**

We searched the Cochrane, PubMed and Embase databases and included original studies focusing on the criterion validity, construct validity and responsiveness of the FI when applied in community-dwelling older people. We evaluated the quality of the studies included using the Quality in Prognosis Studies (QUIPS) tool. This systematic review was conducted based on the PRISMA statement.

**Results:**

Of the twenty studies identified, eighteen reported on FIs derived from research data, one reported upon an FI derived from an administrative database of home-care clients, and one reported upon an FI derived from routine primary care data. In general, the FI showed good criterion and construct validity but lacked studies on responsiveness. When compared with studies that used data gathered for research purposes, there are indications that the FI mean score and range might be different in datasets using routine primary care data; however, this finding needs further investigation.

**Conclusions:**

Our results suggest that the FI is a valid frailty screening instrument. However, further research using routine Electronic Medical Record data is necessary to investigate whether the psychometric properties of the FI are generalizable to a primary care setting and to facilitate its interpretation and implementation in daily clinical practice.

**Trial registration:**

PROSPERO systematic review register number: CRD42013003737.

## Background

Among other issues, ageing within the population poses a major burden on healthcare due to the increasing prevalence of frailty among older people [1]. Frailty is defined as a state of increased vulnerability due to somatic, environmental or psychosocial factors [2]. To better accommodate for the complex care needs of frail, older people, a transition towards proactive, population-based care is required, which will improve clinical outcomes and cost-effectiveness [3,4]. To facilitate this care transition, general practitioners (GPs) must be capable of identifying frail older patients within their daily clinical practice.

The Frailty Index (FI) is one of the screening tools for frailty [5]. An FI comprises a list of health deficits (e.g. symptoms, signs, impairments, and diseases) that are indicative of frailty. The proportion of deficits present forms the patient’s FI score, which can range from zero to one [6]. When an FI consists of at least 30 deficits, different numbers and types of deficits may be used without major influence on the properties of the FI, which enables application in and comparison between different datasets [7].

There is considerable debate over whether the FI can be used for frailty screening in daily primary care. Some authors have stated that the FI has not been validated in this setting, that the instrument is of limited value due to its perceived complexity, that the FI has only moderate discriminative ability, and that other frailty instruments, such as the Tilburg Frailty Indicator, are more promising [8-11]. Others have argued that the FI is a significant predictor of adverse health outcomes, that it covers all important frailty factors, that it can be easily derived from routine administrative healthcare data, and they have called for further exploration of the FI’s merits in primary care [12-14].

To further assess the potential of the FI as a screening and monitoring instrument for frailty in primary care, knowledge of its characteristics is essential. Therefore, we performed a systematic review of the literature and assessed the psychometric properties of the FI in identifying frailty among community-dwelling older people.

## Methods

### Search strategy, selection criteria and data extraction

We searched the Cochrane, PubMed, and Embase databases using the terms ‘frailty AND (index OR deficit OR deficits OR cumulative OR accumulation)’. We searched for studies published from August 8^th^, 2001 onwards, which is the publication date of the landmark study presenting the FI concept [6]. The search was limited to studies in English, and databases were searched until October 30^th^, 2012. The first and third author (ID and GK) screened titles and abstracts independently and selected studies for full-text assessment. These full-text studies were assessed by the first author for inclusion, and in cases where doubt existed, an independent assessment by the last author (MS) followed. Citations from the included articles were also searched for additional relevant publications by the first author. Eligibility disagreements were resolved by consensus.

Studies were included that met the following criteria: first, the studies focused on an FI. The FI was defined as a list of health deficits for which patients were screened and that provided an FI score that reflected the proportion of deficits present on the predefined list [6]; second, only original research was included that assessed one of the following psychometric properties of the FI: criterion validity, construct validity or responsiveness; third, the studies focused primarily on community-dwelling older people. Community-dwelling older people were defined as older people who lived independently at home; older people who lived at home while receiving home care; and older people living in assisted living facilities. In the Netherlands, GPs provide care to older people in all these different living situations, and virtually all older people in these living situations are inscribed in a general practice. Studies were excluded when the FI was based on a comprehensive geriatric assessment (CGA), because it is not feasible to perform a CGA for all older patients in general practice. Also, studies were excluded when the entire study population was living in a nursing home, was hospitalized or was selected because of one specific disease in common. Secondary reports of FI datasets that did not report additional psychometric properties were excluded (see Additional file 1 for full details of inclusion and exclusion criteria). Based on these predefined criteria, the first author extracted data on general study characteristics, frailty index characteristics and assessed psychometric properties.

### Psychometric properties– definitions

Currently, there is no consensus about a frailty reference standard against which the criterion validity of the FI could be assessed. However, since there is general agreement that the concept of frailty reflects a state of increased vulnerability to adverse health outcomes, criterion validity is defined as the ability of an FI to predict adverse health outcomes [15]. An Area Under the Curve (AUC) of < 0.70 was considered poor; an AUC of 0.70-0.89 was considered adequate; and an AUC of ≥ 0.90 was considered excellent [16]. Construct validity refers to the coherence of the FI with other frailty measures or related conditions and constructs, including comorbidity, disability, self-rated health, age, and gender [15]. Responsiveness reflects the ability of the FI to detect clinically important changes over time in the frailty construct (see Additional file 1 for a detailed description of the various psychometric properties) [17].

### Quality assessment

Study quality was evaluated using the Quality in Prognosis Studies (QUIPS) tool, which considers six potential domains of bias: inclusion, attrition, prognostic factor measurement, confounders, outcome measurement, and analysis and reporting [18]. Each domain comprises a number of prompting items, which enable assessment of the domain as having a high, moderate or low risk of bias.

The QUIPS tool was considered the most appropriate quality appraisal tool because, conceptually, the frailty index is a prognostic instrument. We modified three domains of the QUIPS tool. First, in our review, we were interested only in the descriptive, rather than explanatory, relationships of the FI to adverse health outcomes and other measures; thus, we considered the domain ‘confounders’ irrelevant. Second, the domain ‘outcome measurement’ only accommodated studies in which the FI correlated with adverse outcomes, i.e., criterion validity studies. We modified this domain such that the QUIPS tool also applied to studies in which the FI was correlated cross-sectionally or longitudinally with other frailty measures or related constructs, i.e., construct validity or responsiveness studies. Third, in the domain ‘prognostic factor measurement’, we redefined the prompting item ‘Valid and Reliable Measurement of Prognostic Factor’ as ‘Valid and Reliable Construction of Prognostic Factor’ because the FI deficit list must be constructed based on specific criteria [2,19]: first, deficits should be acquired and related to health status; thus, ‘blue eyes’ is not an appropriate deficit whereas ‘heart failure’ is appropriate; second, deficit prevalence should increase with age; third, deficits should not ‘saturate’ too early, for example, presbyopia is present in almost all older people, thus, it is not appropriate as a deficit; fourth, the combination of deficits in an FI should cover a range of systems; fifth, the same FI should be used in follow-up measures; and finally, the FI should comprise at least 30 deficits and deficit prevalence should be at least 1% [2] (see Additional file 2 for the modified QUIPS form that was used for the quality appraisal of the studies included).

### Registration

This systematic review was registered prospectively in the PROSPERO international prospective register of systematic reviews (CRD42013003737).

### Funding

This research was supported by a grant from ZON-MW, The Netherlands Organization for Health Research and Development (reference 311040201). The sponsor had no influence on the research design, data collection, data interpretation, the writing of this report or the decision to publish.

## Results

### Search results

**Figure 1:**
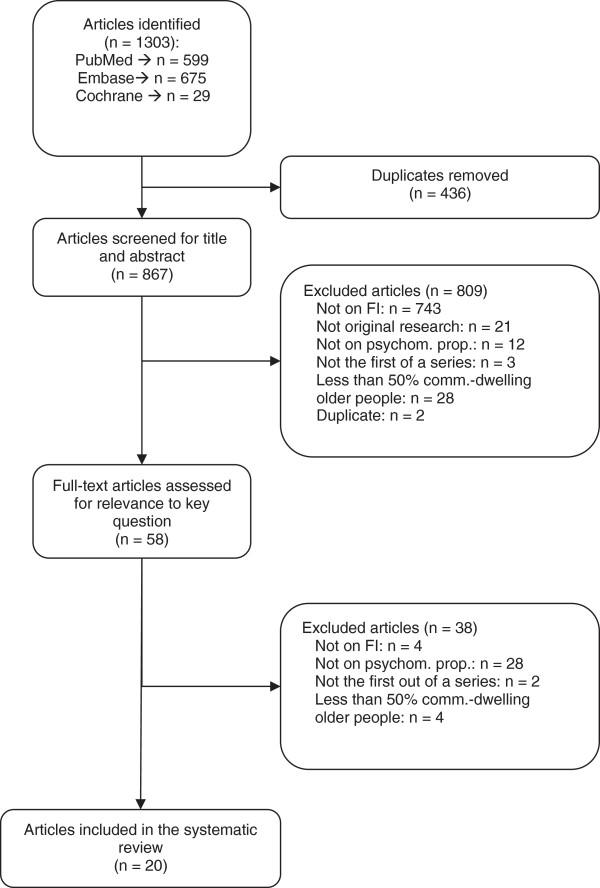
Flowchart of search results.

After removing duplicates, our search resulted in 867 studies (Figure 1). We excluded 809 studies after screening the titles/abstracts and 38 studies after full-text assessment. We have listed the full bibliographic details and the reason for exclusion of each of these studies (available upon request). No additional studies were found in manual reference searching; thus, we used twenty studies for our final review.

### Description of study characteristics

One study was a cross-sectional study [20], and nineteen studies were cohort studies with a follow-up ranging from one to twelve years (Table 1). Eighteen studies used survey datasets; one study used an administrative dataset of home-care clients [21], and one study was based on the analysis of routine administrative primary care data [22].

**Table 1:** General characteristics of the studies included

***Study***	***Design data set***	***Total N (% women)***	***Follow-up (LTFU)***	***FI deficits***			***FI scores***	
		***Mean age (yrs ± SD)***		***Deficit number***	***Deficit scoring***	***Deficit weighting***	***Mean/Median *** **(± SD/IQR)**	***Range***
		***Setting***						
**Armstrong et al. **[21]	Retrospective cohort study	23,952 (69.4%)	1 yr (?)	50	B	No	?	? – 0.66
		81.7 (± 7.4)						
	8 CCACs	Home-care clients						
**Cigolle et al. **[20]	Cross-sectional study	1,657 (55.5%)	N/A	38	?	?	?	?
		?						
	HRS	Community-dwelling						
**Drubbel et al. **[22]	Retrospective cohort study	1,679 (59%)	2 yrs (10.5%)	36	B	No	0.08 (0.03-0.14)	0 – 0.42
		Median 73 (IQR 65–81)						
	GPs EMRs	Community-dwelling						
**Fang et al. **[32]	Retrospective cohort study	3,257 (51.1%)	8 yrs (13.8%)	33	B/M	No	0.13 (± ?)	0 – 0.67
		70.1 (± 9.0)						
	BLSA	Community-dwelling						
**García-González et al. **[33]	Retrospective	Total sample: 4,872	1.95 yrs (13.2%)	34	B/M	No	0.16 (± 0.11)	0 – 0.65
	cohort study	Analyzed sample:						
	MHAS	4,082 (52.5%)						
		73 (range 65–105)						
		Community-dwelling						
**Gu et al. **[23]	Retrospective cohort study	13,861 (57.2%)	3 yrs (12.9%)	39	B	Yes	0.26 (± ?)	?
		? (range 65–109)						
	CLHLS	Population-based						
**Hogan et al. **[37]	Retrospective cohort study	1,066 (76.7%)	1 yr (0%)	83^a^	B/M	No	?	?
		84.9 (± 7.3)						
	ACCES	Assisted living residents						
**Kulminski et al. **[24]	Retrospective cohort study	4,721 (?%)	4 yrs (0%)	48	B	No	?	0 – 0.70
		?						
	CHS	Population-based						
**Kulminski et al. **[25]	Retrospective cohort study	24,206 (65.9%)	4 yrs (?)	32	B	No	0.25 (± ?)	0 – 0.70
		78.3 (± ?)						
	NLTCS	Population-based						
**Lucicesare et al. **[27]	Prospective cohort study	1,016 (55.4%)	4 yrs (0%)	43	B	No	0.14 (± ?)	0 – 0.70
		74.7 (± 7.1)						
	CSBA	Population –based						
**Lucicesare et al. **[28]	Retrospective cohort study	1,318 (63.1%)	5 yrs (?)	38	?	?	?	0 – 0.59
		76.05 (± ?)						
	CSHA	Population-based						
**Mitnitski et al. **[34]	Retrospective cohort study NPHS, CSHA (3), ALSA, SOPSA, NHANES, H-70, NLTCS-I, ICONS, BCS	36,424 (58.5%)	3-12 yrs (?)	10 FI’s: 38-40	B/M	No	?	?
		74 (range: 27 – 105)						
		7 community-dwelling and 4 clinical/institutional samples		1 FI: 13				
**Mitnitski et al. **[6]	Retrospective cohort study	2,913 (?%)	5 yrs (?)	92	B	No	?	?
		82 (± 7.4)						
	CSHA	Population-based						
**Rockwood et al. **[28]	Retrospective cohort study	2,305 (?%)	5 yrs (?)	70	B/M	No	?	0 – 0.70
		?	?					
	CSHA	Population-based						
**Searle et al. **[9]	Retrospective cohort study	754 (64.6%)	9 yrs (<10%)	40	B/M	No	?	0 – 0.60
		?						
	YPEP	Community-dwelling						
**Shi et al. **[35]	Retrospective cohort study	3,257 ((51.1%)	8 yrs (12.2%)	35	B/M	No	?	? – 0.70
		70.1 (± 9.0)						
	BLSA	Community-dwelling						
**Song et al. **[29]	Retrospective cohort study	2,740 (60.8%)	10 yrs (10.1%)	36	B	No	0.15 (± ?)	0 – 0.70
		74 (± 6.6)						
	NPHS	Population-based						
**Theou et al. **[36]	Retrospective cohort study	2,305 (62.1%)	5 yrs (?)	FI 1: 37^b^	B/M	No	FI 2: 0.24 (± 0.15)	0 – 0.68
		84.6 (± 7.0)		FI 2: 37^c^				
	CSHA	Community-dwelling						
**Woo et al. **[30]	Prospective cohort study	4,000 (50%)	4 yrs (15.9%)	47	B	No	?	?
		?						
	CUHKS	Community-dwelling						
**Woo et al. **[31]	Retrospective cohort study	2,032 (50.8%)	10 yrs	62	B	Yes	0.13 (?)	0 – 0.53
		?	42.4% (3 yrs)					
	HKHS	Population-based	85.3% (10 yrs)					

? = no information found/unclear; ^a^In this study, two FIs were assessed: the Armstrong index and the Full Frailty Index. Only the second FI is reported here (both FIs show similar results); ^b^Excluding ADLs/comorbidities, ^c^Including ADLs/comorbidities comprising 37 different deficits to FI 1; B = binary scoring; FI = Frailty Index; IQR = Interquartile range; LTFU = Lost to follow-up; M = multilevel scoring; N/A = not applicable; Population-based = representative sample of community-dwelling and institutionalized older people; SD = standard deviation; Data sources: ACCES = Alberta Continuing Care Epidemiological Studies; ALSA = Australian Longitudinal Study of Ageing; BCS = Breast Cancer Survivor Study; BLSA = Beijing Longitudinal Study of Ageing; CCAC = Community Care Access Centre; CHS = Cardiovasculair Health Study; CLHLS = Chinese Longitudinal Healthy Longevity Survey; CSBA = Conselice Study of Brain Ageing; CSHA: Canadian Study of Health and Ageing; CUHKS = Chinese University of Hong Kong Study; GPs EMR = General Practitioners’ Electronic Medical Record; H-70 = Gothenburg Study; HKHS = Hong Kong Health Survey; HKSPH = Hong Kong School of Public Health study; HRS = Health and Retirement Survey; ICONS = Improving Cardiovascular Outcomes in Nova Scotia; MHAS = Mexican Health and Aging Study; NHANES = National Health and Nutrition Examination Survey; NLTCS (−i) = National Long Term Care Survey (−institute); NPHS = National Population Health Survey; SOPSA = Sydney Older Persons Studies on Aging; YPEP = Yale Precipitating Events Project.

In ten studies, the study population was population-based, consisting of a representative mixture of independently living and institutionalized older people, with the majority of people living independently [6,23-31]. Eight studies included only independently living older people [19,20,22,32-36]; and two studies focused specifically on older people receiving home care or older people in assisted living facilities [21,37]. The number of participants ranged from 754 to 36,424 older people with a mean age varying from 70.1 to 84.9 years, and the percentage of women varied from 50.0 to 76.7%.

The FIs used in the studies were based on 13 to 92 health deficits. Most studies scored deficits dichotomously [6,21-26,29-31]. Eight studies applied multilevel scoring [19,28,32-37] and used, for example, a Likert-scale [33]. Two studies did not report how the deficits were scored [20,27]. Two studies assigned extra weight to predefined deficits [23,31], for example, to ‘polypharmacy’ [31]. The mean FI scores varied from 0.13 to 0.26, and except for two studies that reported a lower maximum FI score [22,31], the maximum reported FI score varied from 0.60 to 0.70.

### Quality assessment

Four studies showed a low risk of bias for each of the five domains of the QUIPS tool considered, namely inclusion, attrition, prognostic factor measurement, outcome measurement, and analysis and reporting. Fourteen studies showed a moderate-to-high risk of bias in one or two domains; and two studies showed a moderate-to-high risk of bias in three or four domains (Table 2). Risks of bias were highest in the domain of study attrition, which was due to very low response rates [31] or an unclear response rate [19,25,34]. In one cohort study, attrition was not assessed because only the cross-sectional study component was considered [27]. For the remaining fourteen cohort studies, losses to follow-up were < 16%.

**Table 2:** Assessment of risk of bias using the ‘Quality Assessment in Prognostic Studies’ (QUIPS) tool

***Study***	***Study participation***	***Study attrition***	***Prognostic factor measurement***	***Outcome measurement***	***Statistical analysis***
**Armstrong et al. **[21]	Low	Low	Low	Moderate	Low
**Cigolle et al. **[20]	Low	N/A	Moderate	Low	Moderate
**Drubbel et al. **[22]	Low	Moderate	Moderate	Low	Low
**Fang et al. **[32]	Low	Moderate	Moderate	Low	Low
**García-González et al. **[33]	Low	Moderate	Low	Low	Low
**Gu et al. **[23]	Low	Low	Low	Low	Low
**Hogan et al. **[37]	Low	Low	Low	Low	Low
**Kulminski et al. **[24]	Moderate	Low	Moderate	Low	Low
**Kulminski et al. **[25]	Low	High	Low	Low	Low
**Lucicesare et al. **[26]	Low	Low	Moderate	Low	Moderate
**Lucicesare et al. **[27]	Low	N/A^a^	Moderate	Low	Low
**Mitnitski et al. **[34]	Low	High	Moderate	Low	Low
**Mitnitski et al. **[6]	Low	Moderate	Low	Low	Low
**Rockwood et al. **[28]	Moderate	Moderate	Low	Low	Low
**Searle et al. **[19]	Low	High	Moderate	Low	Low
**Shi et al. **[35]	Low	Low	Low	Low	Low
**Song et al. **[29]	Low	Low	Low	Low	Low
**Theou et al. **[36]	Low	Moderate	Moderate	Low	Moderate
**Woo et al. **[30]	High	Moderate	Moderate	Low	Moderate
**Woo et al. **[31]	Low	High	Moderate	Low	Low

Low = low risk of bias, Moderate = moderate risk of bias, High = high risk of bias. Level of risk of bias was determined by judgement of the prompting items belonging to each assessed domain. ^a^Attrition was not assessed because only the cross-sectional component in which construct validity was examined was of interest.

In the domain of prognostic factor measurement, eleven studies were judged as having a moderate risk of bias [19,20,22,24,27,28,30-32,34,36]. Of these eleven studies, four studies did not report their entire FI deficit list [20,26,27,32], three used data-driven cut-off points for the FI [24,26,30], and nine did not report the percentage of missing FI data or how missing FI data were managed [19,20,22,24,30-32,34,36]. In the remaining nine studies showing a low risk of bias in the prognostic factor measurement, eight reported a percentage of missing data of <5% [21,23,25,28,29,33,35,37], and one study did not report the percentage of missing data [6]. Six studies managed missing data by excluding the missing deficits from the denominator when calculating the FI [6,25,28,32,35,37]. Two studies imputed the missing FI data [23,29]. All twenty studies complied with the criteria for adequate FI construction as described in the ‘Methods’ section.

In total, in the 20 included studies, 5.1% of domains, i.e., inclusion, attrition, prognostic factor measurement, outcome measurement, and analysis and reporting as assessed with the QUIPS tool showed a high risk of bias, 25.5% of domains showed a moderate risk of bias, and 69.4% of domains showed a low risk of bias (full QUIPS appraisal forms for each study are available upon request).

### Psychometric properties of the FI

#### Criterion validity

Fifteen studies assessed the criterion validity of the FI by evaluating the predictive ability of the FI for mortality, institutionalization, hospitalization, number of days in hospital, morbidity, Emergency Department (ED) visits, out-of-hours GP consultations, falls, fractures, change in ADL score, and change in mental score (Table 3). In each study, the FI was incorporated into a multivariable regression model that was corrected for age, gender and a variety of other co-variables. In each model, the FI was a significant predictor of the assessed outcome.

**Table 3:** Criterion validity results; the predictive ability of the frailty index for adverse health outcomes

***Study***	***Outcome variable with events (n)***	***Model***	***Factors controlled for in model***	***Effect measure***	***95*% *CI/SE***	***Interpretation effect measure***
**Armstrong et al. **[21]	Mortality: 1676	Cox proportional hazards regression	Age, gender	FI: HR = 1.93	1.79-2.08	Most frail (15%) vs. least frail (60%) group
	Institutionalization: 4550			(EFS: HR = 2.49)	(2.32-2.68)	
				(CHESS: HR = 2.32)	(2.15-2.51)	
**Drubbel et al. **[22]	Mortality/ED visits/institutionalization/out-of-hours GP surgery visits: 508	Cox proportional hazards regression	Age, gender, consultation gap	HR = 1.166	1.129-1.210	Per deficit increase in FI score
**Fang et al. **[32]	Recurrent falls: 109	Logistic regression	Age, gender, education	OR = 1.54	1.34-1.76	Per one-unit increment in FI score
	Recurrent fractures: 174	Logistic regression	Age, gender, education	OR = 1.07	0.94-1.22	Per one-unitincrement in FI score
	Mortality: 1101	Cox proportional hazards regression	Age, gender, education, falls, fractures	HR = 1.29	1.25-1.33	Per one-unit increment FI score
**García-González et al. **[33]	Mortality: 279	Cox proportional hazards regression	Age, gender	HR = 6.45	4.10-10.14	Most frail (FI 0.35-0.65) vs. least frail group (0.00-0.07)
**Gu et al. **[23]	Mortality: 5,753	Weibull proportional hazards regression	Age, ethnicity, urban–rural residence, SES, family/social connection and support, health practices	Men (65–79):		Most frail vs. least frail quartile
				HR = 4.56	0.96	
				Women (65–79):		
				HR = 3.84	1.01	
**Hogan et al. **[37]	Mortality: 170	Logistic regression	Age, gender, co-morbidity	RR = 2.35	1.56-3.54	All analyses: most frail (FI > 0.30) vs. least frail group (FI < 0.20)
	≥ 1 hospitalization: 424	Logistic regression	Age, gender, co-morbidity	RR = 1.28	1.04-1.57	
	Institutionalization: 204	Logistic regression	Age, gender, co-morbidity	RR = 3.30	2.29-4.76	
**Kulminski et al. **[24]	Mortality: 421	Cox proportional hazards regression	Age, gender, FP	FI: RR = 1.035	1.026-1.045	
				(FP: RR = 1.014)	(1.009-1.019)	Per 1% increment in FI score (or FP)
**Kulminski et al. **[25]	Mortality: 2146	Cox proportional hazards regression	Age, gender	RR = 1.029	1.001	Per 1% increment in FI score
**Lucicesare et al. **[26]	Mortality: 147	Cox proportional hazards regression	Age, gender, CSBA score	FI: HR = 5.26	1.05-26.42	?
				(CSBA score: HR = 1.52)	(1.28-1.81)	
**Mitnitski et al. **[34]	Mortality (%/yr) 3.7-20.6	Cox proportional hazards regression	Age, gender	CSHA-s: HR = 1.031	0.003	Per deficit increase in FI score
				CSHA-c: HR = 1.054	0.007	
				CSHA-i: HR = 1.046	0.009	
				SOPSA: HR =1.079	0.022	
				NHANES: HR = 1.011	0.003	
**Searle et al. **[19]	Mortality: ?	Cox proportional hazards regression	Age, gender	HR = 1.03	1.02-1.04	Per 0.01 increase in FI score
**Shi et al. **[35]	Mortality: 1,155	Cox proportional hazards regression	Age, gender	HR = 1.13	1.09-1.47	Per deficit increase in FI score
**Song et al. **[29]	Mortality: 1,208	Cox proportional hazards regression	Age, gender	FI: RR = 1.57	1.41-1.74	Per FI level (FI ≤ 0.08; FI between 0.08-0.25; FI ≥ 0.25).
**Theou et al. **[36]	Mortality: 1002	Cox proportional hazards regression	Age, gender, nr. of ADL disabilities, nr. of chronic diseases	FI 1: HR = 1.11	1.06-1.17	Per 0.1 increase in FI score
**Woo et al. **[31]	Change in ADL score 0–3 yrs^a^	Linear regression	Age, gender, ADL score at baseline	B = −4.99	−7.68 - −2.30	Per 1.0 increase in FI score
	Change in mental score 0-3 yrs^a^	Linear regression	Age, gender, mental score at baseline	B = −2.23	−4.11 - −0.35	Per 1.0 increase in FI score
	Change in hospital days 0–3 yrs^a^	Linear regression	Age, gender, hospital days at baseline	B = 45.74	28.16 – 63.33	Per 1.0 increase in FI score
	New diseases at three yrs^a^	Ordinal logistic regression	-	For FI = 0.00, predicted probability ≥ 1 new disease = 17.4%		Predicted probabilities for new diseases at 3 years
				For FI = 0.50, predicted probability ≥ 1 new disease = 52.2%		

^a^Regression models with 3-year outcomes reported due to excess LTFU at 10 years. 95% CI = 95% Confidence Interval; adm. = admission; ADL = Activities of Daily Living; B = beta; CHESS = Changes in Health, End-Stage Disease and Signs and Symptoms Scale; CSBA = Conselice Study of Brain Ageing; CSHA = Canadian Study of Health and Ageing; DI = Deficit Index (Frailty Index); EFS = Edmonton Frail Scale; FI = Frailty Index; FP = Frailty Phenotype; HR = hazard ratio; NHANES = National Health and Nutrition Examination Survey; OR = odds ratio; PBA = Personal Biological Age; RR = relative risk; SE = standard error; SOPSA Sydney Older Persons Studies on Aging.

Twelve studies focused on the prediction of mortality, for which hazard ratios of 1.01 (SE ± 0.003; per deficit increase in the frailty index) to 6.45 (95% CI 4.10-10.14, most-frail group (FI score 0.35-0.65) versus the least-frail group (FI score < 0.07) were reported [34,33]. A multivariable model with age, gender, co-morbidity and an FI resulted in an Area Under the Curve (AUC) of 0.691 (95% CI 0.648-0.733) for one-year mortality [37]. Used as a single independent variable, the FI predicted two-year mortality with an AUC of 0.780 (± 0.020 SE) and a ten-year mortality with an AUC of 0.720 (± 0.020 SE) [29].

For other outcome measures, comparable AUCs were as follows: 0.610 (95% CI 0.576-0.644) for one-year hospitalization risk and 0.667 (95% CI 0.625-0.707) for a one-year risk of moving to long-term care [37]. For the prediction of time to the combined outcome of ED/out-of-hours GP surgery visits, nursing home admission and mortality, the c-statistic of the FI used as a single independent variable was 0.686 (95% CI 0.664-0.708). When the FI was combined in a model with age, gender, and consultation gap, the c-statistic improved to 0.702 (95% CI 0.680-0.724) [22].

One study tested the added value of the FI in a multivariable model for predicting adverse health outcomes. For mortality and transition to long-term care, the AUCs of the models including an FI were significantly higher than the AUCs of a model comprising only age, gender and co-morbidity (p < 0.03). For hospitalization, the AUC of the full model with age, gender, co-morbidity and an FI was significantly higher than the AUC of a model comprising only age and gender (p < 0.001) [37].

#### Construct validity

Eleven studies evaluated the construct validity of the FI [6,20,21,24-28,34,36,37]. The FI showed a strong positive correlation with the Functional Reach test (r = 0.73) [29], Consolice Study of Brain Ageing (CSBA) score (r = 0.72) [26], Frailty Phenotype (0.65) [28], and Edmonton Frail Scale (EFS; r = 0.61) [21], a strong negative correlation with the Mini Mental State Examination score (r = −0.58) [28], and a moderate correlation with the Changes in Health, End-Stage Disease and Signs and Symptoms (CHESS) Scale (r = 0.35) [21]. When the dichotomized FI was compared with the Frailty Phenotype where the latter was used as a reference standard, the FI showed a sensitivity of 45.9 to 60.7% and a specificity of 83.5 to 90.0% [20,24]. When compared with the Functional Domains model, the sensitivity of the FI was 38%, and its specificity was 91.5% [20]. When using a three-level risk categorization, the weighted kappa of the FI compared with the Frailty Phenotype was 0.17 (95% CI 0.13-0.20), and the weighted kappa of the FI compared with the CHESS scale was 0.36 (95% CI 0.31-0.40).

The FI displayed moderate correlation with the concept of self-rated health (r = 0.49), which was expressed as an index of self-rated health deficits [27]. When the crude correlation of the FI was assessed with age, a weak to moderate correlation of 0.193, 0.241 and 0.320, respectively, was reported [6,25,26]. One study compared the age trajectories of the FI score within community-dwelling and institutional/clinical cohorts [34], with higher levels of comorbidity and disability in the latter. The FI score increased gradually with age in community-dwelling cohorts, whereas the FI score was high at all ages in the institutional/clinical cohorts.

One study examined specifically an FI with only symptoms and signs as deficits and demonstrated that older people with higher FI scores showed more functional impairments in (I) ADL and more co-morbidity than patients with lower FI scores [36].

Without formally assessing correlations within a construct validity context, sixteen studies reported that older people and women show higher FI scores [6,19,20,22,23,25-37], and only one study reported a lower percentage of women in the most-frail group [21].

Six studies quantified the increase in FI score with chronological age, all reporting a similar increase in FI score with age ranging from +0.02 to 0.05/year [6,19,22,26,34,35].

No studies reported on the responsiveness of the FI in daily clinical practice.

## Discussion

In this systematic review, we demonstrate that the FI adequately predicts a wide range of adverse health outcomes and that its discriminative capability is poor to adequate. The FI correlates strongly with other frailty measures, except for the CHESS scale. However, this scale is not a frailty measure per se but was designed to measure ‘health instability’ and to specifically predict mortality in institutionalized older people [38]. The FI score increases steadily with age, and the maximum FI score reported was 0.70, indicating that no ceiling effect exists.

Our review has a number of strengths. First, we used a broad, sensitive search strategy with a low risk of missing relevant studies. Thus, we identified a large number of studies with consistent results across a variety of FIs in different populations. Second, we only considered relevant psychometric properties. We omitted reliability because the FI is an automated screening procedure and therefore not susceptible to intra- or interrater variability. Internal consistency was not examined because the FI is a formative model, i.e., the items form the construct together and therefore do not need to be correlated [39]. Third, the definitions used were tailored specifically to those aspects considered essential for frailty measures and based on a standardized taxonomy [15,17]. Fourth, we tailored our detailed inclusion and exclusion criteria to support our aim, which was to select those FI studies relevant for primary care. For example, we excluded studies with an FI based on a comprehensive geriatric assessment because it is not feasible to perform such an assessment for each older patient in primary care. Fifth, we appraised included studies critically using the QUIPS tool, which provided comprehensive quality assessment that demonstrated overall good quality of the methodology used in the included studies. The majority of studies reported sufficient details on their study sample, used appropriate criteria for FI construction, and reported few missing data. Moreover, the reported loss to follow-up was typically well below 20%; thus, biased results were unlikely [40].

Our review also has several limitations. First, there is a risk of publication bias because studies with negative results are less likely to be published [41]. Because no register exists for validation studies, publication bias could not be formally assessed. Second, due to the withdrawal of one of the authors (GK), the first author (ID) performed the full-text assessment and quality appraisal partially alone, which may have caused potential selection bias. However, strict predefined selection and quality appraisal criteria were applied (see Additional files 1 and 2), and in cases where doubt existed, full-texts were assessed independently by the last author (MS). Third, most of the included studies on construct validity lacked prespecified hypotheses, which increases the risk of bias because, retrospectively, alternative explanations for low correlations may be sought [39]. Because the majority of correlations were robust, this risk appears limited. Finally, an individual patient data meta-analysis would have been preferable when summarizing research on the criterion validity of the FI. However, because the nature and number of deficits differed between the studies, it was not feasible to merge these data. Moreover, due to study heterogeneity, a meta-analysis on the outcome measures was not possible [41].

Apart from the FI, another frailty screening instrument that has shown good criterion and construct validity is the Frailty Phenotype [42]. One may question whether this performance-based measure would be preferable to implement in general practice, since it has also good face validity, consisting of five easily interpretable parameters (unintentional weight loss, self-reported exhaustion, weakness, slow walking speed, and low physical activity). However, compared to the FI, the Frailty Phenotype would require extra time and resources to enable execution in daily clinical care, and in direct comparison, the FI has been shown to better predict mortality risk among older people [24].

Our results are consistent with previous FI reviews that also reported on criterion validity and construct validity of the FI [7,13,43]. Our review updates these findings, and whereas these previous reviews were narrative in nature, our review is the first to systematically review the FI’s psychometric properties that are relevant to primary care.

In the majority of the included studies on the FI’s criterion validity, its predictive ability for mortality is examined. This does not mean that the FI is meant to be a ‘mortality prediction’ instrument; rather, by including the FI in a multivariable model including age, the FI score aims to explain the variable vulnerability to adverse health outcomes in people of the same age. This heterogeneity in frailty levels is also reflected by the relatively low correlation coefficients that we found between FI and age; whereas, in general, the correlation coefficient for the mean FI scores versus age was high (e.g. r = 0.985, [34]), the correlation coefficient for the individual FI scores versus age was at maximum 0.320 [26].

To assess the construct validity of the FI, we focused on its correlation with other frailty measures, age, gender, disability, comorbidity, and self-rated health [15]. However, the concordance of the FI with a broad array of other measures has also been investigated, and a high FI score has been demonstrated to correlate with a high and low BMI [44], smoking [45,46], impaired psychological well-being [47], psychiatric illness [48], impaired mobility [49], impaired cognition and Alzheimer’s disease [50,51], pain [52], high levels of gonadotropins [53], neighborhood deprivation and low individual socio-economic status [54], rural residence [55,56], and low education and little social support or participation [57]. The FI may also serve as a basis to calculate ‘biological age’. Individuals with an FI score that is relatively high for their age and gender show a biological age that is higher than their chronological age, and this biological age is also a significant predictor of mortality [58].

There is no evidence supporting responsiveness or utility. However, some studies reflected upon the potential utility of the FI and noted two major advantages: first, the FI can be constructed from available data whether from administrative routine primary care data [22], specific measurements, such as the interRAI-AL instrument [37], or comprehensive geriatric assessment data [26,29]. Second, the FI score can be calculated using software thereby facilitating its clinical application [24,37]. However, only in one study the FI was actually studied in routine clinical data, so these potential advantages need to be further explored.

One may argue that studies relating FI score change to baseline factors, such as mobility and baseline frailty state, and studies modeling FI score change [49,59] do describe responsiveness. These studies demonstrate that FI score development over time can be adequately described using a time dependent Poisson distribution, and that the probability of improvement, stability and worsening of the FI score is directly related to the baseline number of deficits, age, and mobility status. However, we did not consider these studies as responsiveness studies, since they did not study pre-specified hypotheses regarding the expected correlations between changes in the score on the FI instrument, and changes in other variables, such as scores on other instruments, or demographic or clinical variables [17]. An important finding of our systematic review is that eighteen out of twenty studies explored the FI’s psychometric properties in datasets gathered specifically for research purposes. These studies consistently showed a higher maximum and mean FI score compared with the study that investigated the FI using routine primary care data [22]. however, because only one study with an FI using routine primary care data was included, there is not enough evidence to support conclusions about any structural differences in mathematical properties of the FI. More FIs applied in routine primary care data sets should be studied to further explore these potentially different mathematical properties. The narrower FI score range in the study using routine primary care data reflects unexpectedly low deficit prevalences, which may be caused by several reasons: first, patients may experience symptoms or problems with which they do not present themselves to the GP; second, there may be suboptimal data registration in the EMR [60,61], and third, the FI may need to include more items on level of functioning, mobility or health attitude instead of merely relying on morbidity deficits. Also, except for the polypharmacy deficit, this FI was based on one single data source out of the Electronic Medical Records (EMRs), namely symptoms and diagnoses encoded according to the International Classification of Primary Care (ICPC, [62]). Care should be taken to construct an FI that captures all information available in the EMR by using, for example, not only ICPC-encoded data but also diagnostic measurement data, such as body mass index or laboratory tests, and elaborate medication data, encoded according to the Anatomic Therapeutic Chemical (ATC) [63].

## Conclusions

In this systematic review, the FI demonstrates good criterion and construct validity, but its discriminatory ability is poor to moderate. In general, the FI appears to be an easily interpretable instrument that is practical to manage; however, studies that focus on its responsiveness, interpretability or utility are lacking. These results support the potential of the FI as a screening instrument for frailty in primary care and also demonstrate that further research into its psychometric properties is required. FIs based on research data show lower FI scores than those based on routine primary care data. Given its implementation in clinical practice, future validation studies of the FI should focus primarily on its application in routine primary care data.

## Competing interests

The authors declare that they have no competing interests.

## Authors’ contributions

ID, MJS, MEN and NJW contributed to the study concept and design. ID drafted the manuscript. ID and GK selected relevant studies independently, and ID extracted data and assessed the quality of the included studies. ID, NJW, NB, MEN and MJS provided a critical review of this manuscript. All authors read and approved the final version of this manuscript.

## Pre-publication history

The pre-publication history for this paper can be accessed here:

http://www.biomedcentral.com/1471-2318/14/27/prepub

## Supplementary Material

Additional file 1 Systematic review on the psychometric properties of the frailty index. Click here for file

Additional file 2 QUIPS risk of bias assessment instrument for prognostic factor studies. Click here for file
