# Supplementary material for: Screening for frailty in primary care: a systematic review of the psychometric properties of the frailty index in community-dwelling older people
Source: BMC Geriatr. 2014 Mar 6;14:27. doi: 10.1186/1471-2318-14-27 (PMC3946826; doi:10.1186/1471-2318-14-27)
Supplement: Additional file 1 — Systematic review on the psychometric properties of the frailty index. [file 1471-2318-14-27-S1.docx]

**Systematic Review on the Psychometric Properties of the Frailty Index**

**Eligibility form**

**1.** Is it a study about the frailty index?

No

**2.** Is it original research?

No

**1.** Exclude

Yes

**2.** Exclude

Yes

**3.** Is it a study that focuses on criterion validity, construct validity, or responsiveness?

**3.** Exclude

Yes

**5.** Is this the only study using this frailty index in this data set, is it the first study of a series using this frailty index in this same data set, or a later one that adds additional information on psychometric properties compared to the first study?

No

No

**5.** Exclude

Yes

No

**4.** Exclude

**INCLUDE**

**4.** Does (>50% of) the study population consist of community-dwelling older people, who are not specifically selected because they all have the same disease?

Yes

**Additional information on used eligibility criteria**

1. The frailty index concept as proposed by Mitnitski and Rockwood should be the main focus of the article, or should be one of multiple frailty measures that are compared. In the original concept, the frailty index consists of a list of health deficits. Patients are screened for those deficits, and the resulting frailty index score is the proportion of deficits present out of the predefined list. A frailty index based on a comprehensive geriatric assessment is excluded, because in primary care, performing a CGA for all older patients would not be feasible.
2. All original research should continue to step 3, irrespective whether it is a cross-sectional, observational, case-control study or trial. Examples of articles not considered as original research are reviews, letters, editorials, and commentaries.
3. The following definitions of psychometric properties are used:
   1. *Criterion validity^1^* exists when a new definition or test correctly classifies people according to a referent outcome. The outcome can either be an accepted test of impeccable validity or the prediction of an outcome. No frailty referent standard exists yet, but one means of testing the criterion validity of a definition of frailty would be to assess its ability to predict adverse outcomes. *Example:* predictive ability of the frailty index for death or institutionalization.
   2. *Construct validity^1^* refers to whether the operational definition coheres with other measures of the phenomenon, related conditions and constructs. Construct validity is typically measured by correlation of the new definition with like measures. We focus on correlation with other frailty measures, disability, co-morbidity, self-rated health, age, and gender. Construct validity studies examining relations of the FI with other measures than the above mentioned should be excluded.
   3. *Responsiveness^2^* refers to the ability of an instrument to detect clinically important change over time in the construct to be measured. It can be seen as a measure of longitudinal construct validity. Pre-specified hypotheses should have been formulated concerning expected mean differences between changes in groups or expected correlations between changes in the scores on the instrument and changes in other variables, such as scores on other instruments, or demographic or clinical variables. Furthermore, to quantify whether the instrument distinguishes clinically important change from measurement error, the Smallest Detectable Change (SDC) should be related to the Minimal Important Change (MIC). Another adequate measure is the AUC, which is a measure of the ability to distinguish patients who have and have not changed, according to an external criterion. *Examples:* correlation between change in the frailty index and change in (I)ADL score, difference in change in the frailty index between community-dwelling and institutionalized older people.
4. Older people are defined as people aged 60 years or older. Community-dwelling is defined as living independently with or without home care, or living in an assisted living facility. Studies in an Emergency Department setting, hospital or nursing home should be excluded, just as studies that specifically focus on a study sample in which older people all have the same disease should be excluded.
5. If multiple studies use the same frailty index in the same data set, only the first study should be included, unless later studies add information about the psychometric properties of the frailty index.

*References:*

**1)** Rockwood K. What would make a definition of frailty successful? Age and Ageing 2005:34;432-434.

**2)** [Terwee CB](http://www.ncbi.nlm.nih.gov/pubmed?term=Terwee%20CB%5BAuthor%5D&cauthor=true&cauthor_uid=17161752), [Bot SD](http://www.ncbi.nlm.nih.gov/pubmed?term=Bot%20SD%5BAuthor%5D&cauthor=true&cauthor_uid=17161752), [de Boer MR](http://www.ncbi.nlm.nih.gov/pubmed?term=de%20Boer%20MR%5BAuthor%5D&cauthor=true&cauthor_uid=17161752), [van der Windt DA](http://www.ncbi.nlm.nih.gov/pubmed?term=van%20der%20Windt%20DA%5BAuthor%5D&cauthor=true&cauthor_uid=17161752), [Knol DL](http://www.ncbi.nlm.nih.gov/pubmed?term=Knol%20DL%5BAuthor%5D&cauthor=true&cauthor_uid=17161752), [Dekker J](http://www.ncbi.nlm.nih.gov/pubmed?term=Dekker%20J%5BAuthor%5D&cauthor=true&cauthor_uid=17161752), [Bouter LM](http://www.ncbi.nlm.nih.gov/pubmed?term=Bouter%20LM%5BAuthor%5D&cauthor=true&cauthor_uid=17161752), [de Vet HC](http://www.ncbi.nlm.nih.gov/pubmed?term=de%20Vet%20HC%5BAuthor%5D&cauthor=true&cauthor_uid=17161752). Quality criteria were proposed for measurement properties of health status questionnaires. J Clin Epidemiol. 2007 Jan;60(1):34-42. Epub 2006 Aug 24.
